# Predicting voxel-level dose distributions of single-isocenter volumetric modulated arc therapy treatment plan for multiple brain metastases

**DOI:** 10.3389/fonc.2024.1339126

**Published:** 2024-02-14

**Authors:** Peng Huang, Jiawen Shang, Zhihui Hu, Zhiqiang Liu, Hui Yan

**Affiliations:** Department of Radiation Oncology, National Cancer Center/National Clinical Research Center for Cancer/Cancer Hospital, Chinese Academy of Medical Sciences and Peking Union Medical College, Beijing, China

**Keywords:** multiple brain metastases, volumetric modulated arc therapy, radiotherapy, deep learning, dose prediction

## Abstract

**Purpose:**

Brain metastasis is a common, life-threatening neurological problem for patients with cancer. Single-isocenter volumetric modulated arc therapy (VMAT) has been popularly used due to its highly conformal dose and short treatment time. Accurate prediction of its dose distribution can provide a general standard for evaluating the quality of treatment plan. In this study, a deep learning model is applied to the dose prediction of a single-isocenter VMAT treatment plan for radiotherapy of multiple brain metastases.

**Method:**

A U-net with residual networks (U-ResNet) is employed for the task of dose prediction. The deep learning model is first trained from a database consisting of hundreds of historical treatment plans. The 3D dose distribution is then predicted with the input of the CT image and contours of regions of interest (ROIs). A total of 150 single-isocenter VMAT plans for multiple brain metastases are used for training and testing. The model performance is evaluated based on mean absolute error (MAE) and mean absolute differences of multiple dosimetric indexes (DIs), including (*D*_max_ and *D*_mean_) for OARs, (*D*_98_, *D*_95_, *D*_50_, and *D*_2_) for PTVs, homogeneity index, and conformity index. The similarity between the predicted and clinically approved plan dose distribution is also evaluated.

**Result:**

For 20 tested patients, the largest and smallest MAEs are 3.3% ± 3.6% and 1.3% ± 1.5%, respectively. The mean MAE for the 20 tested patients is 2.2% ± 0.7%. The mean absolute differences of *D*_98_, *D*_95_, *D*_50_, and D_2_ for PTV60, PTV52, PTV50, and PTV40 are less than 2.5%, 3.0%, 2.0%, and 3.0%, respectively. The prediction accuracy of OARs for *D*_max_ and *D*_mean_ is within 3.2% and 1.2%, respectively. The average DSC ranges from 0.86 to 1 for all tested patients.

**Conclusion:**

U-ResNet is viable to produce accurate dose distribution that is comparable to those of the clinically approved treatment plans. The predicted results can be used to improve current treatment planning design, plan quality, efficiency, etc.

## 1 Introduction

Brain metastasis is the cancer that occurs when cancer cells from their original sites spread to the brain. The typical tumor sites causing brain metastasis are the lung, breast, colon, and kidney. Brain metastases could be single or multiple tumor sites in the brain (1, 2). The brain metastases could cause pressure on the brain. Also, the function of the surrounding brain tissue could be changed by the tumor. The symptoms of brain metastases include memory loss, seizures, headaches, etc. (3). The traditional treatment methods for brain metastases are surgery, whole-brain radiotherapy (WBRT), three-dimensional conformal radiation therapy (3D-CRT), hypo-fractionated stereotactic radiotherapy (SRT), and single-fraction stereotactic radiosurgery (SRS) (4–7).

WBRT and 3D-CRT have been traditionally used for the treatment of multiple brain metastases. However, WBRT can cause cognitive dysfunction or dementia, while 3D-CRT takes a long time to treat multiple brain metastases (8–10). In SRS/SRT, a higher accuracy of patient positioning is required. Recently, the developments of image-guided radiotherapy (IGRT) and volumetric modulated arc therapy (VMAT) techniques have provided precise target localization and quick dose delivery for patients under radiotherapy. The introduction of VMAT not only takes a short time in treatment delivery but also shows a highly conformal dose comparable to conventional SRS/SRT (11, 12). The treatment of brain metastases using VMAT has been accepted as a routine treatment modality in recent years (13, 14).

Compared to multiple-isocenter VMAT, single-isocenter VMAT is popular due to its quick and accurate beam delivery for the treatment of multiple brain metastases (15–17). However, to achieve an ideal dose distribution, a set of suitable plan optimization parameters (dose constraints and their weighting factors) is needed prior to the optimization of the treatment plan. Also, planners have to adjust these parameters manually during plan optimization, which usually takes several hours. To address this issue, knowledge-based planning (KBP) was proposed (18, 19) in the last decade. They implemented plan automation through optimization algorithms or templates from previously treated patients. These methods can partially reduce the effort involved in parameter fine-tuning but still require human involvement (20). Recently, the research interest in KBP has transitioned from classic machine learning methods to modern deep learning methods (21–25). Unlike classic machine learning methods, modern deep learning methods can directly learn features from the original data and predict 3D doses with high precision.

The recent development of the dose prediction model is mostly based on the U-Net structure, which consists of an encoder and decoder with skip connections. 2D U-Net was first applied to prostate IMRT plans by Nguyen et al. (21). After that, many efforts were made. Residual learning was introduced to the dose prediction model by several researchers (22–25), while dense connectivity was used to enhance feature representation capability by other researchers in their models (26–28). In addition, other types of networks, such as Resnet (27, 29, 30) and GAN (31–33), are also used for dose prediction. So far, the deep U-net-like architecture and its variants with various types of residual or dense blocks become the mainstream structure for dose prediction (34–38).

With the successful applications of deep learning models in predicting dose distribution for many primary tumor sites such as the lung (25, 26), head-and-neck (23, 28, 33, 34), and prostate (21, 35), it is interesting to investigate this application for brain metastasis. In the study, a deep U-net architecture (30), previously successfully applied to predict dose distribution for head-and-neck cancer patients, is used as the base model in predicting the dose distribution of the VMAT plan for brain metastasis. The rest of this paper is organized as follows: In Methods, the patient data, prediction model, and experimental settings are introduced in detail. In Results, the prediction accuracy of the deep learning model is evaluated by comparing it with the dose distribution of the clinically approved plans. Finally, the advantages and disadvantages of the prediction model are discussed, and future work is prospected in the Discussions.

## 2 Methods

### 2.1 Patient data

The dataset consists of 150 single-isocenter VMAT treatment plans designed for multiple brain metastases patients treated in our institute during 2019–2022. All patient plans are made by medical physicists and approved by radiation oncologists for clinical treatment. The number of tumors in each patient is varied from one to four. PTVs include PTV60 for 31 patients, PTV52 for 41 patients, PTV50 for 34 patients, and PTV40 for 44 patients. Primary OARs include body, brain stem, spinal cord, left lens, right lens, left optic nerve, right optic nerve, and optic chiasm.

The 150 patient plans are randomly divided into three sets: 100 for training sets, 30 for validation sets, and 20 for testing sets. These VMAT plans are designed with two arcs and delivered with 6 MV beam energy. The input images are all rescaled to 256 × 256 × 21 matrixes (7 for CT images, 7 for contour image, and 7 channels for target prescriptions), and the output image is 256 × 256 × 1 matrixes (dose distributions on each slice). This study was conducted in accordance with the Declaration of Helsinki (as revised in 2013). This study was approved by the ethics committee of the National Cancer Center/Cancer Hospital, Chinese Academy of Medical Sciences, and Peking Union Medical College. The committee waived the written informed consent because this is a retrospective study.

### 2.2 Prediction model

**Figure 1:**
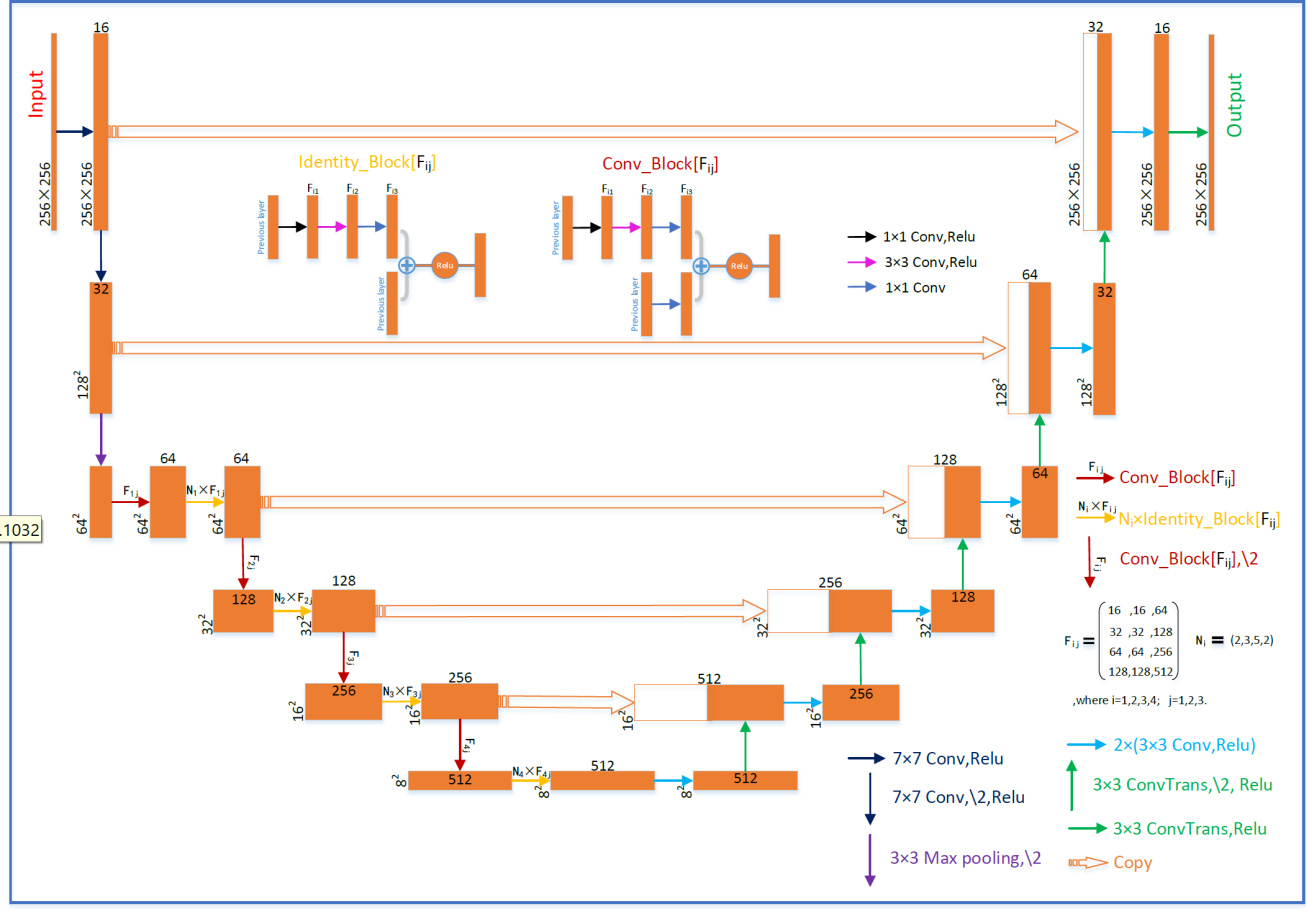
Schematic diagram of the deep U-net architecture.

The U-net with residual network (U-ResNet) model incorporating residual convolutional and de-convolutional blocks is shown in **Figure 1**. It consists of contracting and expansive paths. The contracting path follows convolutional layers and stacked building blocks of Identity-Block and Conv-Block to extract multiscale patient-specific features, doubling the number of feature maps at each step. The expansive path at each step consists of a de-convolutional block that halves the number of feature maps and concatenation with the corresponding feature map from the contracting path. The network ends with one de-convolution with 1 × 1 filters replacing 3 × 3 filters.

In the training and validation process, the training samples are augmented by randomly flipping, rotating, scaling, or shifting. The model is trained from scratch with the layer kernel weights initialized using Xavier uniform initialization. Adam optimizer (39) with a batch size of 4 is used for optimization. The initial learning rate (LR) is 1e−4, and the LR is reduced to 20% of its original value if the validation loss does not improve after 10 epochs. The training process is also stopped if the validation loss does not improve after 20 epochs. The model with the best performance on the validation samples is obtained for testing. The proposed network is implemented in Keras with TensorFlow as the backend on a workstation equipped with two NVIDIA GeForce 2080 Ti GPUs. The training process for a single model takes around 20 h. The prediction process for one case takes less than 1 s.

### 2.3 Model evaluation

The mean absolute error (MAE) is used to evaluate the accuracy of the predicted 3D dose distribution. It is the average error over all voxels of the body and is defined as **Equation 1**:


(1) $${\text{MAE}}_{k}=\frac{1}{N_{k}}\sum_{i=1}^{N_{k}}\left|D_{P}-D_{T}\right|\times 100\%$$

Where *N_k_* is the number of total voxels belonging to the *k*th structure. *D_P_* and *D_T_* are the predicted and ground-truth (or calculated) doses of the *i*th voxel. The voxel doses were normalized by the value of the prescription dose. Several traditional dosimetry indexes (DIs) (*D*_max_, *D*_mean_ for OARs and *D*_98_, *D*_95_, *D*_50_, and *D*_2_ for PTVs), conformity index (CI), and homogeneity index (HI) are also evaluated.

CI formula is defined as **Equation 2**:


(2) $$\text{CI}=\frac{V_{T,\text{ref}}}{V_{T}}\times \frac{V_{T,\text{ref}}}{V_{\text{ref}}}$$

$V_{T,\text{ref}}$ is the volume of the target volume at which the received dose is equal to or greater than the reference dose; *V_T_* is the volume of the target volume; *V*_ref_ is the volume at which the received dose is equal to or greater than the reference dose. The closer the value of CI is to 1, the better the target is covered. HI formula is defined as **Equation 3**:


(3) $$\text{HI}=\frac{D_{2}-D_{98}}{D_{50}}$$

where *D_n_* represents the minimum radiation dose received by *n*% of the volume of the radiation area. The closer the value of HI to 0, the better the uniformity of the target dose. In addition, the absolute differences in DI between predicted and clinically approved plans are evaluated as follows: |δDI| = |DI_clinical_ − DI_Predicted_|.

The dice similarity coefficient (DSC) between dose distributions is also evaluated and defined as **Equation 4**:


(4) $$\text{DSC}\left(\text{A},\text{B}\right)=\frac{2\left|{\text{A}}^{\cap }\text{B}\right|}{\left|\text{A}\right|+\left|\text{B}\right|}$$

where A represents the clinical isodose volume and B denotes the predicted isodose volume.

## 3 Results

### Dose difference

**Figure 2:**
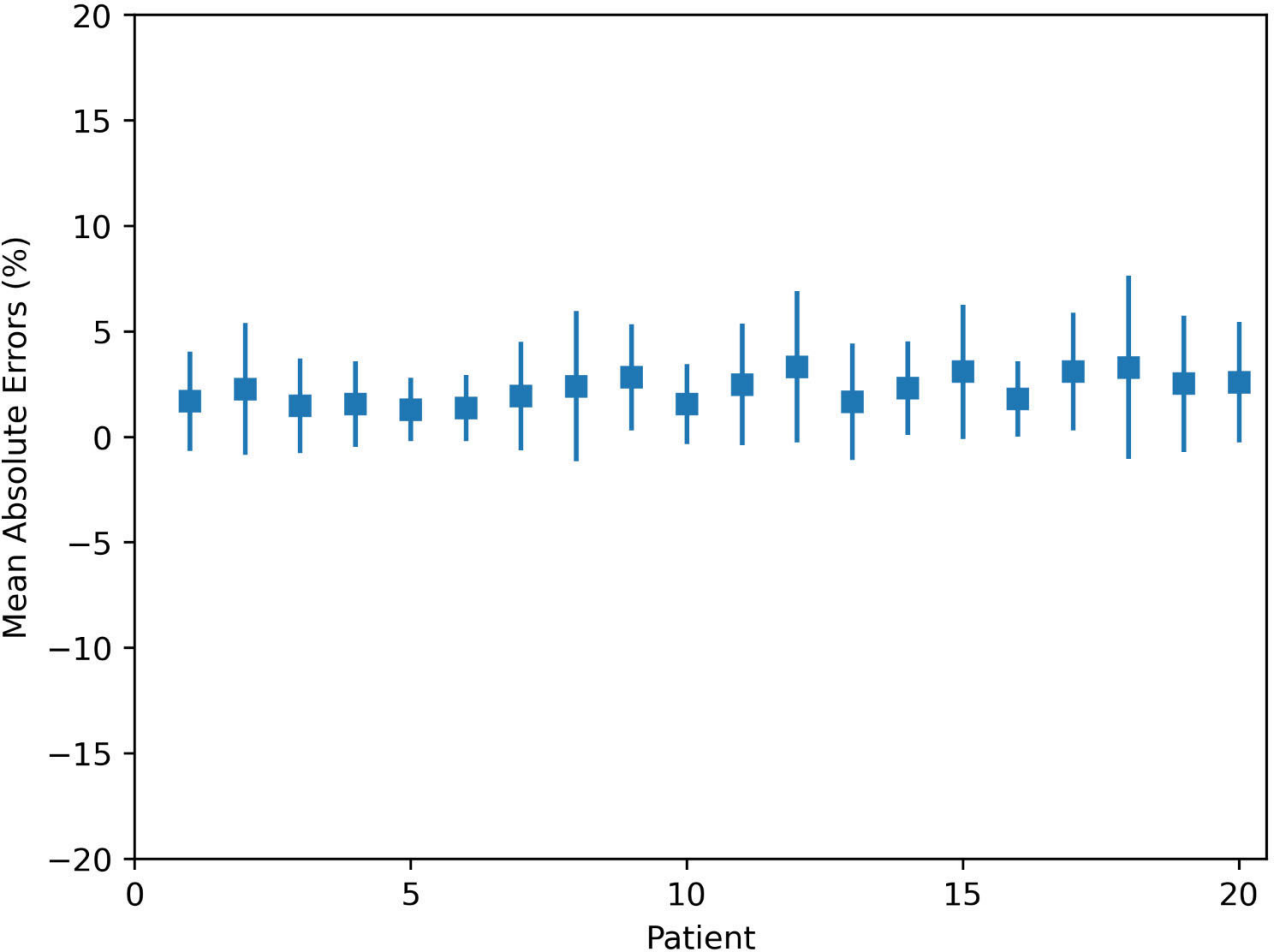
Mean absolute errors within the body for 20 tested patients.

The MAE plot for all 20 tested patients is shown in **Figure 2**. The largest and smallest MAEs are 3.3% ± 3.6% and 1.3% ± 1.5% within the patient’s body, respectively. The largest and smallest MAEs are 5.2% ± 4.0% and 2.1% ± 1.7% within the targets, respectively. The average MAE is 2.2% ± 0.7% (relative to the prescription of PTV) within the body, and the average MAE is 3.6% ± 1.0% within targets.

### Dosimetric index

For PTVs with multiple prescription doses, the dosimetric indexes are shown in **Table 1**. On average, the absolute differences of *D*_98_, *D*_95_, *D*_50_, and *D*_2_ for PTV60, PTV52, PTV50, and PTV40 are less than 2.5%, 3.0%, 2.0%, and 3.0%, respectively. There are no significant differences between predicted and clinically approved plan doses for PTVs. There are no significant differences from the predicted results for HI and CI. Regarding OARs, the dosimetric indexes of *D*_max_ and *D*_mean_ are shown in **Table 2**. The prediction accuracy for *D*_max_ and *D*_mean_ is between 3.2% and 1.2%. Six OARs for *D*_max_ and eight for *D*_mean_ were predicted within 2%. There is no significant difference between clinical and predicted results. For certain patients, the D_max_ and D_mean_ of OARs are close to 0, as they are far from PTV. This causes a large standard deviation of dosimetric results for these OARs. In general, the dosimetric indexes predicted by the model well match those from the clinically approved plans.

**Table 1:** Statistics of dosimetric indexes for PTVs of 20 tested patients.

PTVs	Dosimetric indexes	Clinically approved	Model predicted	|δDI|	*p*-value
PTV60	*D*_98_ (Gy)	60.1 ± 1.0	58.9 ± 1.5	2.1% ± 2.1%	0.054
	*D*_95_ (Gy)	60.7 ± 0.8	59.5 ± 1.2	2.2% ± 1.9%	0.063
	*D*_50_ (Gy)	62.8 ± 1.2	62.4 ± 1.0	1.9% ± 2.0%	0.541
	*D*_2_ (Gy)	65.0 ± 2.3	64.9 ± 1.5	2.5% ± 1.4%	0.947
	HI	0.1 ± 0.0	0.1 ± 0.0	0.0% ± 0.0	0.336
	CI	1.0 ± 0.0	1.0 ± 0.0	0.0% ± 0.0	0.282
PTV52	*D*_98_ (Gy)	50.7 ± 0.7	49.4 ± 1.8	3.0% ± 1.2%	0.142
	*D*_95_ (Gy)	52.2 ± 0.1	51.3 ± 0.9	1.8% ± 1.5%	0.116
	*D*_50_ (Gy)	57.1 ± 0.8	56.1 ± 0.7	2.1% ± 1.4%	0.104
	*D*_2_ (Gy)	60.5 ± 2.1	59.7 ± 1.6	2.0% ± 1.9%	0.271
	HI	0.2 ± 0.0	0.2 ± 0.0	0.0% ± 0.0	0.657
	CI	1.0 ± 0.0	0.9 ± 0.0	0.0% ± 0.0	0.087
PTV50	*D*_98_ (Gy)	49.0 ± 0.6	48.2 ± 0.4	1.4% ± 2.0%	0.489
	*D*_95_ (Gy)	50.3 ± 0.4	49.3 ± 0.3	2.0% ± 1.4%	0.295
	*D*_50_ (Gy)	55.5 ± 0.5	54.7 ± 0.2	1.5% ± 0.7%	0.208
	*D*_2_ (Gy)	59.4 ± 0.8	59.4 ± 0.6	0.2% ± 0.2%	0.627
	HI	0.2 ± 0.0	0.2 ± 0.0	0.0% ± 0.0	0.391
	CI	1.0 ± 0.0	0.9 ± 0.0	0.0% ± 0.0	0.353
PTV40	*D*_98_ (Gy)	38.3 ± 0.6	37.9 ± 0.4	1.0% ± 1.2%	0.207
	*D*_95_ (Gy)	40.0 ± 0.1	39.4 ± 0.8	1.6% ± 1.6%	0.225
	*D*_50_ (Gy)	43.3 ± 1.1	43.6 ± 0.3	2.6% ± 1.6%	0.741
	*D*_2_ (Gy)	45.9 ± 1.6	46.5 ± 0.4	3.0% ± 1.1%	0.400
	HI	0.2 ± 0.0	0.2 ± 0.0	0.0% ± 0.0	0.453
	CI	1.0 ± 0.0	0.9 ± 0.0	0.0% ± 0.0	0.350

**Table 2:** Statistics of dosimetric metrics for OARs in 20 tested patients.

	Dmax (Gy)				Dmean (Gy)			
OARs and body	Clinically approved	Model predicted	|δDI| (%)	*p*-value	Clinically approved	Model predicted	|δDI| (%)	*p*-value
Brain stem	4.7 ± 4.8	4.2 ± 4.9	2.8 ± 2.1	0.247	1.1 ± 1.1	1.1 ± 1.6	0.9 ± 1.0	0.990
Spinal cord	0.7 ± 1.7	0.7 ± 1.6	0.6 ± 1.3	0.961	0.1 ± 0.2	0.0 ± 0.1	0.1 ± 0.2	0.113
Center lens	0.6 ± 0.8	0.8 ± 1.0	0.7 ± 0.7	0.076	0.4 ± 0.7	0.6 ± 0.9	0.6 ± 0.5	0.219
Right lens	0.8 ± 0.9	0.7 ± 0.8	0.6 ± 0.6	0.450	0.5 ± 0.7	0.4 ± 0.5	0.6 ± 0.5	0.248
Center optic nerve	1.7 ± 3.0	1.4 ± 2.5	0.9 ± 0.9	0.161	1.1 ± 2.1	1.0 ± 1.9	0.7 ± 0.6	0.302
Right optic nerve	1.3 ± 1.5	1.1 ± 1.6	1.2 ± 1.1	0.233	0.7 ± 1.0	0.6 ± 1.1	0.8 ± 0.8	0.508
Optic chiasm	3.7 ± 4.5	3.8 ± 6.0	1.8 ± 3.2	0.857	1.3 ± 2.0	1.2 ± 2.0	1.2 ± 1.4	0.789
Body	60.2 ± 7.3	59.4 ± 7.5	3.2 ± 2.5	0.137	1.6 ± 1.1	1.6 ± 1.0	0.3 ± 0.2	0.705

**Figure 3:**
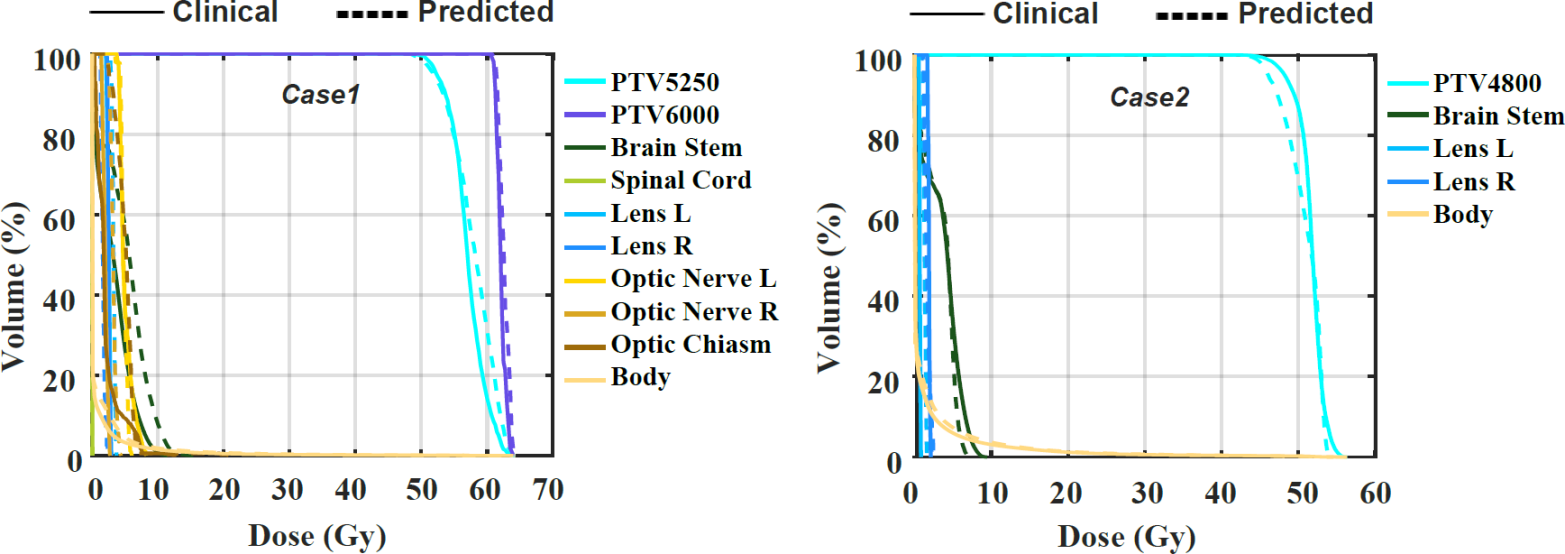
The comparison of the clinical and predicted DVHs for two cases.

The examples of two patients’ DVHs are presented in **Figure 3**. The clinical and predicted DVHs are shown in solid and dashed lines, respectively. Case 1 has two prescription doses (5,250 cGY and 6,000 cGy) and more OARs, while case 2 has one prescription dose (4,800 cGy) and three OARs. For OARs, the maximal dose discrepancy is presented in the higher dose region of the brain stem in both cases. For PTV, the maximal dose discrepancy is presented in the higher dose region of PTV5250 in case 1 and the lower dose region of PTV4800 in case 2.

### Volumes similarity

**Figure 4:**
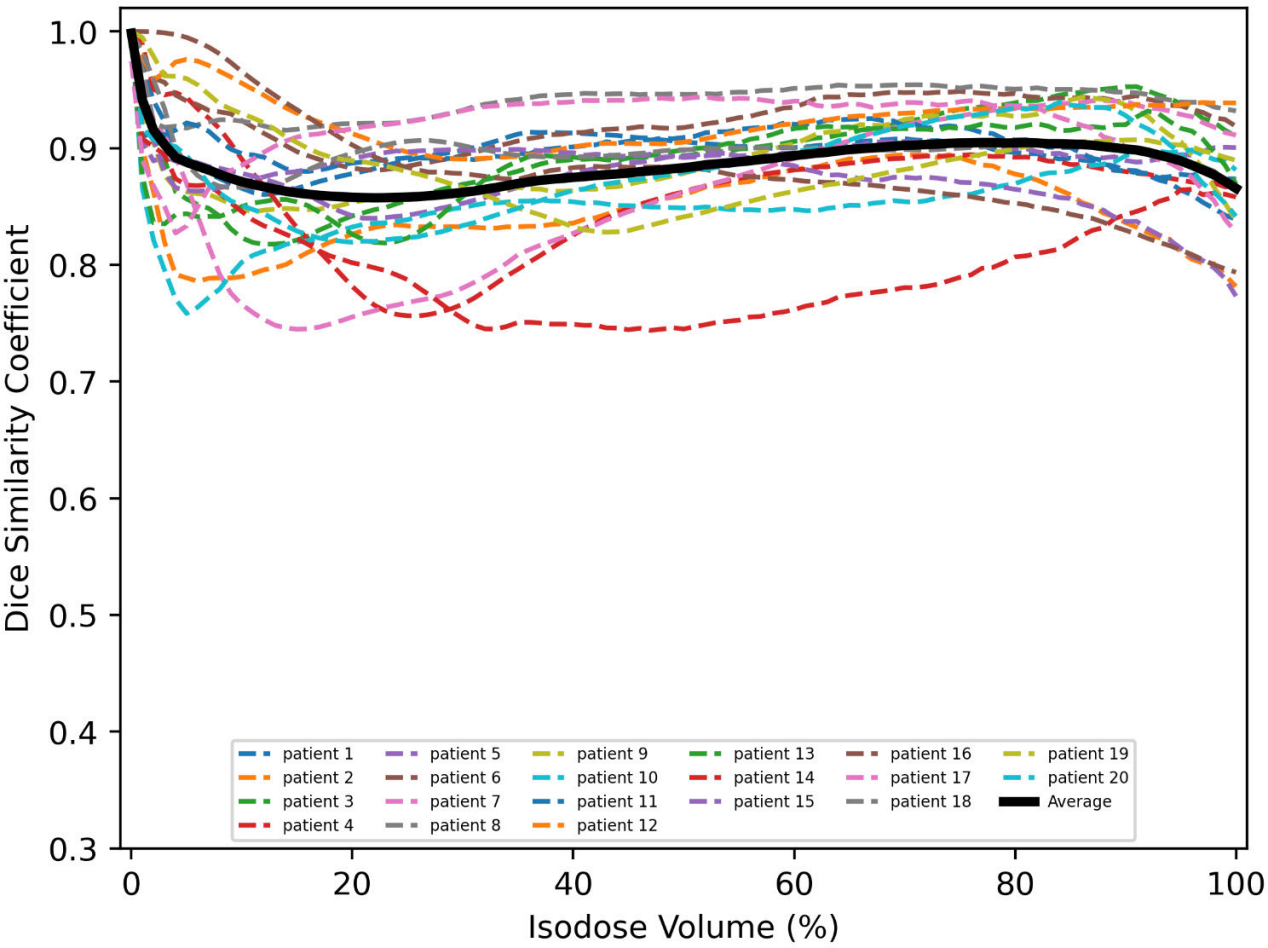
Similarity between clinical and predicted isodose distributions for 20 tested patients.

The dice similarity coefficients between predicted and clinically approved plan doses for the different isodose volumes are calculated. As shown in **Figure 4**, the DSC versus isodose volumes for 20 tested patients are presented. The black curve denotes the averaged DSC curve, which usually ranges from 0 to 1, with 1 standing for ideal match. The averaged DSC for the different isodose volumes ranges from 0.86 to 1.

**Figure 5:**
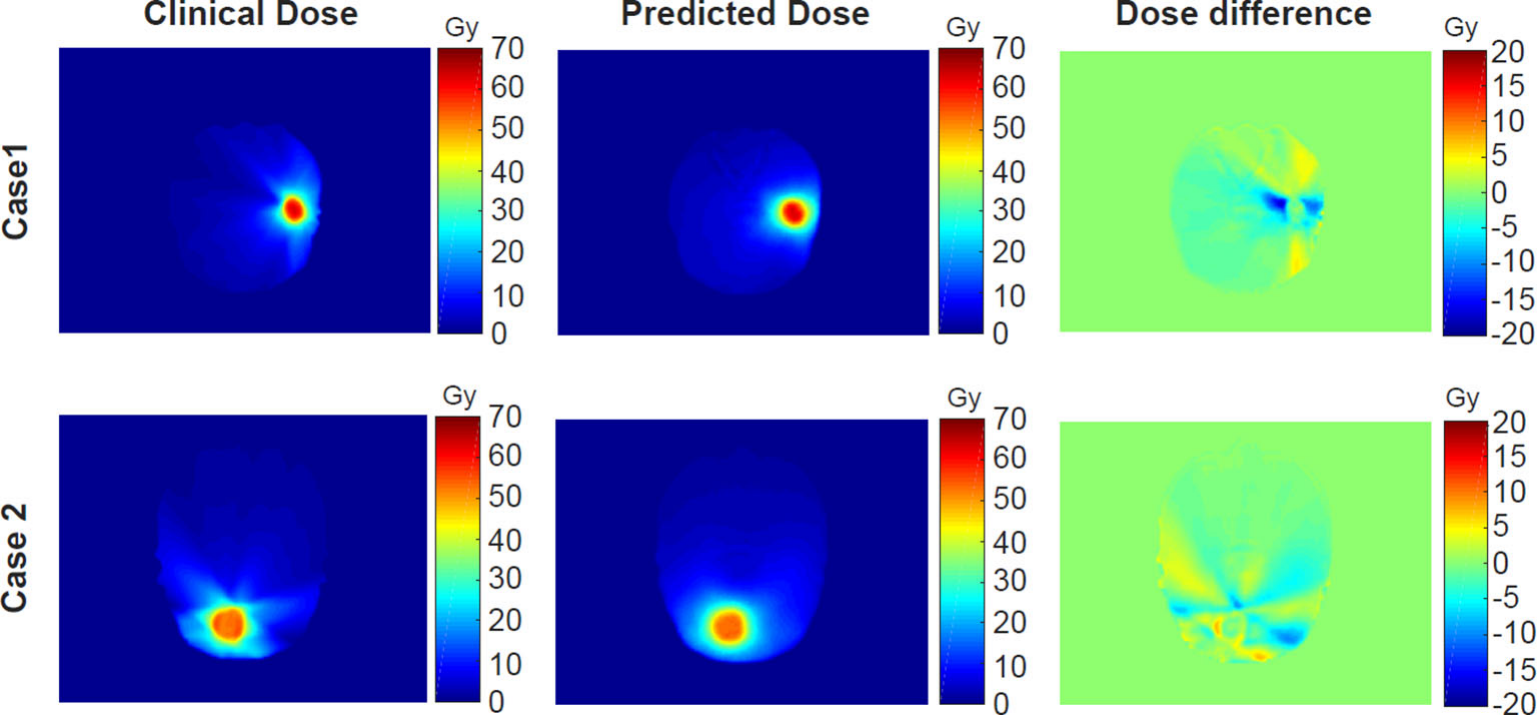
The comparison of the clinical and predicted dose maps for two cases.

Corresponding to the cases shown in **Figure 3**, their clinical and predicted dose maps in 2D slices are presented in **Figure 5**. In the first and second columns, the clinical and predicted dose maps in axial view are displayed with a color wash pattern. The different images between the first and second columns are presented in the third column. For case 1, the predicted doses are higher than the clinical doses in two small regions on the left and right sides of PTV. For case 2, the predicted doses are less than the clinical doses on the left-bottom sides of PTV. Overall, the predicted and clinical doses are highly consistent.

## 4 Discussions

In this study, an advanced deep learning model is applied to predict 3D dose distribution based on our clinical dataset. As far as we know, there is no deep learning model used in predicting the dose of VMAT plans for multiple brain metastases. Using 150 brain metastases from VMAT plans, the U-ResNet model exhibits accurate dose distribution and high efficiency. As shown in **Table 1**, the mean prediction errors range from 1.9% to 2.5%, 1.8% to 3.0%, 0.2% to 2.0%, and 1.0% to 3.0% for PTV60, PTV52, PTV50, and PTV40, respectively. For the absolute value of the PTV dose, the mean value of the predicted plan doses is slightly less than that of the clinically approved plan doses. This may be due to the inclusion of various target prescriptions in a single model, where the varying combinations of prescriptions may impact the prediction accuracy of the target dose. The limited number of samples and larger variation of tumor sites may be another reason. There is no significant difference in dosimetric indexes between clinically approved and predicted plan doses. Although the results demonstrate that the prediction accuracy is acceptable for clinical use, there is still a certain room for improvement.

Although U-ResNet succeeded in dose prediction, as reported by many researchers, there is still a lot of room for improvement. The receptive field would be enlarged increasingly by the stacked multiple convolution layers in the decoder. However, the network’s capability to catch features in multiscale resolution could be limited. The predicted voxel dose is affected not only by the neighboring voxels but also by the spatial distribution between PTVs and OARs. Thus, to extract multiscale features from the image simultaneously, the introduction of pyramid blocks is needed. We will test the model with the modules in a serial or parallel manner in the future, which could further improve the performance of the prediction model.

There are several challenges to this study. First, it is difficult to collect hundreds of VMAT plans with similar locations and shapes of tumor mass for model learning. In the case of multiple brain metastases, the number of tumor masses and their locations could vary considerably among patients. The limited number of samples and larger variation of tumor sites and shapes will make it hard to learn a solid pattern for a learning model. A more effective model or strategy is needed in dealing with such situations for multiple brain metastases. Second, the introduction of U-ResNet increases the complexity and time of model training. As tested, the time on model training is about 20 h on a workstation equipped with two NVIDIA GeForce 2080Ti GPUs. In the future, we plan to further fine-tune the basic 3D model and build a more memory-efficient mechanism for higher performance.

## 5 Conclusions

In this work, we evaluated a deep-learning model for 3D voxel-by-voxel dose prediction. It is capable of producing accurate dose distribution of VMAT plans for multiple brain metastases. As an improvement over the single U-Net or ResNet, it is a powerful model that can automatically correlate ROI voxel with dose voxel to achieve high-precise 3D dose prediction. The predicted results can be used to improve current treatment planning design, plan quality, and efficiency.

## Data availability statement

The original contributions presented in the study are included in the article/supplementary material. Further inquiries can be directed to the corresponding authors.

## Author contributions

PH: Conceptualization, Methodology, Writing – original draft. JS: Methodology, Software, Validation, Writing – original draft. ZH: Data curation, Formal Analysis, Validation, Writing – review & editing. ZL: Funding acquisition, Project administration, Resources, Writing – review & editing. HY: Conceptualization, Funding acquisition, Supervision, Writing – review & editing.
